# Long-term treatment with alendronate increases the surgical difficulty during simple exodontias – an in vivo observation in Holtzman rats

**DOI:** 10.1186/1746-160X-8-20

**Published:** 2012-07-26

**Authors:** Nicolau Conte-Neto, Alliny de Souza Bastos, Luis Carlos Spolidorio, Rosemary Adriana Chierici Marcantonio, Elcio Marcantonio Jr

**Affiliations:** 1 Department of Diagnosis and Surgery, Division of Periodontology, UNESP- Univ. Estadual Paulista, School of Dentistry, Rua Humaitá, 1680, Araraquara, SP, 14801-903, Brazil; 2 School of Dentistry, Department of Physiology and Pathology, Division of Pathology, UNESP - Univ. Estadual Paulista, Rua Humaitá, 1680, Araraquara, SP, 14801-903, Brazil

**Keywords:** Bisphosphonates, Tooth extraction, Osteonecrosis

## Abstract

**Background:**

Atraumatic teeth extractions protocols are highly encouraged in patients taking bisphosphonates (Bps) to reduce surgical trauma and, consequently, the risk of jaws osteonecrosis development. In this way, this paper aims to report the findings of increased surgical difficulty during simple exodontias in animals treated with bisphosphonates.

**Methods:**

Sixty male Holtzman rats were randomly distributed into three groups of 20 animals and received daily subcutaneous administration of 1 mg/kg (AL1) or 3 mg/kg (AL3) of alendronate or saline solution (CTL). After 60 days of drug therapy all animals were submitted to first lower molars extractions under general anesthesia. Operatory surgical time and the frequency of teeth fractures were measured as principal outcomes and indicators of surgical difficulty degree.

**Results:**

Animals treated with alendronate (AL1 and AL3) were associated to higher operatory times and increased frequency of teeth fractures compared to match controls.

**Conclusions:**

The bisphosphonate therapy may be associated with an increased surgical difficulty and trauma following simple exodontias protocols, which is considered a critical issue when it comes to osteonecrosis development.

## Background

Tooth Extraction is one of the most common procedures in oral surgery practice and the difficulty to perform this procedure varies according to a sort of risk factors. Among these factors, the increased bone density has been recognized as a relevant feature [1,2] that can be an aging physiologic issue or be resulted of antiresorptive drugs, including bisphosphonates (Bps) [3].

Bps have been widely used to stabilize bone loss occasioned by bone disorders, such as osteoporosis and Paget disease (Rogers *et al.* 2000). In this way, it has been generating a great concern due to the increasing number of Bisphosphonate-related Osteonecrosis of the Jaws (BRONJ). Considering the strong correlation between the etiology of this bone disease with tooth extractions [4-6] many efforts, have been made to reduce the surgical trauma in these patients [7,8]. In this context this paper aims to report the findings of increased surgical difficulty, based on the analysis of operatory time and teeth fractures frequency, associated with bisphosphonate therapy.

## Material and methods

### Animals

Sixty male Holtzman rats weighting 155 to 200 g were used and randomly distributed into three groups of 20 animals each. The rats were housed in polypropylene cages in groups of five animals per cage, at controlled room temperature (23 ± 20°C), humidity (55 ± 10%), and 12/12 h light/dark cycle beginning at 7:00 a.m. Standard chow and tap water were available *ad libitum.* All the protocols described here were approved by local Ethics Committe of the School of Dentistry of Araraquara, São Paulo, Brazil (Protocol number 18/2009).

### Treatments

The animals received daily subcutaneous doses of alendronate (Ale, 1 or 3 mg/kg; ALCON, São Paulo, Brazil), or saline solution (0.9% NaCl; control group). After 60 days of alendronate or saline solution treatment, all animals were submitted to lower first molars extractions under general anesthesia using an intraperitoneal injection of ketamine (0.1 ml/100 g body weight).

### Teeth extraction

Teeth extractions were performed by the same operator with the same technique in all animals. Initially, the rats were placed in a dorsal position and fixed in a special device. After that, the surrounding gingival was carefully detached from the lower first molars with a dental explorer and teeth luxation were made using a Hollenback Carver followed by the tooth removal with a forceps, adapted around the cervical line of the tooth.

### Difficulty factors risk indicators

The assessment of extraction difficulty degree was based on teeth fractures frequency and on the time spent to perform the extraction. This last parameter was defined as the interval between the utilization of the first instrument required to the tooth extraction until the use of the last instrument. Extraction time was measured using a digital timepiece for each case included in the study. The same individual (A.S.B.) measured all extraction times [9] to reduce possible bias. Furthermore, both individuals (operator and A.S.B.) were blinded to the study groups’ assessment.

Teeth fractures frequency were defined as a complete loose in tooth continuity involving crown and/or roots [10].

### Statistical analysis

The data were evaluated using the GraphPad Prism 5.0 software package (GraphPad Inc., San Diego, CA USA). The normality of the data was assessed by the Kolmogorov-Smirnov test. Comparisons among groups were performed using the chi-squared test to teeth fractures frequency analysis and Kruskal–Wallis followed by Dunns post test to surgery duration evaluation (non-parametric data). Results are presented as frequency of teeth fractures. Statistical significance was set at 5% with 95% confidence intervals.

## Results

**Figure 1:**
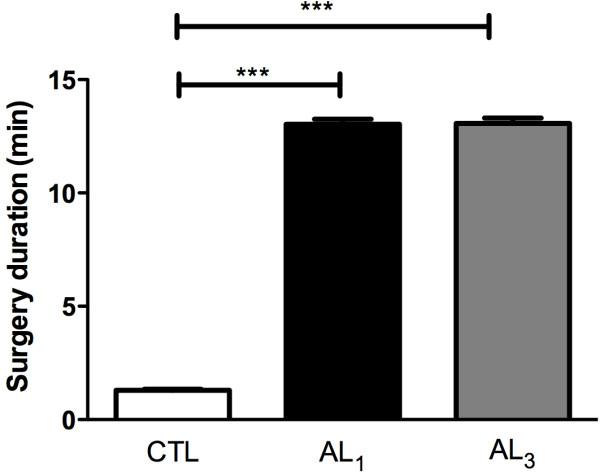
Surgery duration in experimental groups (***p<0.001; Kruskal–Wallis followed by Dunns post test).

During lower first molars extractions of experimental animals, surgical difficulty was markedly increased in animals treated with alendronate 1 mg (AL_1_) and 3 mg (AL_3_) when compared to control group (CTL). Group AL_1_ and AL_3_ animals presented a higher operatory time when compared to animals in control group (Figure 1).

**Figure 2:**
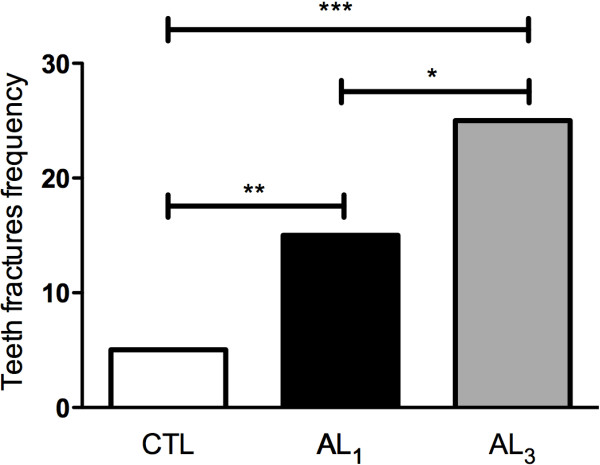
**Teeth fractures frequency in experimental groups.** A total of 40 teeth extractions were performed for each group (*p<0.05; **p<0.01; ***p<0.001; chi-squared test).

Moreover, in the teeth fractures frequency analysis it was observed a significant difference between the groups. While animals in CTL group presented 8% of teeth fractures, animals in alendronate group presented 39.4% (AL_1_) (p<0.01) and 62.5% (AL_3_) (p<0.001). Thus, comparing AL_1_ with AL_3_ groups, it was observed that AL_3_ animals presented teeth fractures frequency significantly increased compared with AL_1_ group (p<0.05) (Figure 2).

## Discussion

In fact, the assessment of surgical difficulty is a relevant issue in the field of oral surgery since allows health professionals to plan operations more accurately helping to minimize surgical trauma, and risks of accidents and complications. This concern is highly relevant to patients treated with bisphosphonates due to the jaws osteonecrosis risk.

The assessment of extraction difficulty has been measured via a wide range of variables [9] and, among of them, the extraction time [11,12] and complications frequency [13,14] are well recognized indicators. For this reason, we selected these two variables as the outcome measures to evaluate the relationship of bisphosphonate therapy with surgical difficulty.

Our findings demonstrated that teeth extractions in animals treated with bisphosphonate require more surgical time when compared to control animals following simple extraction technique. In our opinion, the main reason to justify this surgical time difference is related to the presence of an increased bone density and consequently decrease bone elasticity, that are well recognized difficult factors to teeth extractions [1,2] and are a result of Bps treatment [3].

Even with the absence of bone density evaluation and measurement methods, it is reasonably to believe that Bps treated animals presented a high bone density that is supported by the following points:

1- It is known that the bone effect of Bps is cumulative and assumes a bone absorption linear aspect until 5 mg/kg endovenously dosages [15]. Therefore, the long-term treatment of Bps in high dosages (1 and 3 mg/kg) used in this study resulted in an expressive Bps bone effect.

2- Bps were administered by subcutaneous route that are as effective as endovenously route regarding to drug bioavailability [15]. By this route it is estimated that more of 50% of the drug is available for bone matrix incorporation [16, 17].

3- Due to the high bone turnover in cortical alveolar bone is believed that, although controversial, the Bps bone absorption is higher when compared to other skeletal sites [18], which can be justify by the alveolar lamina dura sclerosis seen in Bps treated patients with BRONJ in initial stage [19]. Besides, the mandibular bone has by itself a higher tissue degree of mineralization when compared to maxillae, been more prone to Bps effects and naturally increases surgical difficulty [9].

In this way, when it is opted to a simple exodontias technique, there is a highly dependence of the tooth to expand the bone tooth socket walls to allow its avulsion and in situations of an alveolar bone increased density there is a lack of sufficient socket expansion which obviously limits the teeth avulsion axis. Consequently, as it happened in this study, requires more surgical manipulation, thereby prolonging operating time [9], as well as the surgical trauma and increases the risk of accidents and complications.

Teeth fractures have been considerate the most frequent accidents during exodontias, in oral surgery practice [20]. They are usually related to inadequate instrumental use and excessive force use, which was one of the reasons that could justify the high frequency of teeth fractures in animals treated with Bps observed in this paper. The concern about this issue is that many efforts have been made to reduce the surgical trauma during teeth extractions by using atraumatic protocols in Bps treated patients [7,8] since exodontias have been considerate as one the most frequent trigger factor to BRONJ [4-6].

In this context, as stated previously, in situations of an increased surgical difficulty degree there is a tendency to prolong surgical length and increase tissue trauma which in field of ONJ can lead to relevant implications:

1- Increase the inflammation of the alveolar bone [21], which could act in favor of the BRONJ lesions development according to the inflammatory theory [22].

2- Result in delayed extraction wound healing due to the compression of bone lining the socket impairing vascular penetration and results in thrombosis of the vessels [21], which could act in favor of the BRONJ lesions development according to the angiogenic theory [23].

3- Increase the risk of dento-alveolar fractures, since when bone tissue becomes too highly mineralized, it also becomes brittle [24]. Moreover, it also makes the tissue more prone to microcrack initiation [25], which act in favor to the BRONJ lesions development according to the bone suppression theory [26].

Another concern that can be discussed regarding to the teeth fractures is about the approaches after these accidents:

1- If opted to extract the residual fragment, the surgical time can be prolonged and increases the tissue trauma, being sometimes necessary to perform bone removal techniques, which can contribute to BRONJ lesions as stated previously.

2- If opted to keep the residual fragment and follow the patient, the surgical trauma will be obviously lower; however, eventually tooth or bone fragments/remnants can lead to an increase in the risk of socket infection [21], which could also increase the risk of osteonecrosis according to the infectious theory [26].

Considering that the more atraumatic is the teeth extraction the better is for the healing process, with special mention in Bps treated patients, we highlight the strategies that can reduce the force intensity and the risk of teeth fracture during the exodontias, such as odontotomy techniques. Extractions without tooth sectioning might be responsible for a more traumatic and difficult surgery, especially in light of difficulty factors, such as increased bone density that can lead to several complications related to BRONJ lesions.

## Conclusions

The bisphosphonate therapy may be associated with an increased surgical difficulty and trauma following simple exodontias protocols, which is considered a critical issue when it comes to osteonecrosis development.

## Consent

A copy of the written consent form is available for review by the Editor-in-Chief of this journal.

## Competing interests

The authors declare that they have no competing interests.

## Authors’ contributions

NCN performed the surgical procedures. ASB helped with the methodological parameters. LCS, RACM and EMJ drafted the manuscript and reviewed it critically. All authors read and approved the final version of the manuscript.

## Authors’ information

NCN is a PhD student from Implantology program at Araraquara School of Dentistry and ASB is a PhD student from Periodontology program at Araraquara School of Dentistry. LCS is a professor and the chairman of the Department of Physiology and Pathology, Division of Pathology at Araraquara School of Dentistry. EMJ and RACM are professors and chairmen of the Department of Diagnosis and Surgery, Division of Periodontology at Araraquara School of Dentistry.
